# Extracellular Vesicles Derived From *Talaromyces marneffei* Yeasts Mediate Inflammatory Response in Macrophage Cells by Bioactive Protein Components

**DOI:** 10.3389/fmicb.2020.603183

**Published:** 2021-01-08

**Authors:** Biao Yang, Jingyu Wang, Hongye Jiang, Huixian Lin, Zihao Ou, Amir Ullah, Yuneng Hua, Juanjiang Chen, Xiaomin Lin, Xiumei Hu, Lei Zheng, Qian Wang

**Affiliations:** ^1^Center for Clinical Laboratory, Zhujiang Hospital, Southern Medical University, Guangzhou, China; ^2^Department of Laboratory Medicine, Nanfang Hospital, Southern Medical University, Guangzhou, China; ^3^Guangdong Engineering and Technology Research Center for Rapid Diagnostic Biosensors, Nanfang Hospital, Southern Medical University, Guangzhou, China; ^4^Shunde Hospital, The First People’s Hospital of Shunde, Southern Medical University, Foshan, China

**Keywords:** *Talaromyces marneffei*, extracellular vesicles, macrophage cells, inflammatory response, proteomics

## Abstract

Extracellular vesicles (EVs) loaded with proteins, nucleic acids, membrane lipids, and other virulence factors could participate in pathogenic processes in some fungi such as *Cryptococcus neoformans* and *Candida albicans*. However, the specific characteristics of EVs derived from *Talaromyces marneffei* (TM) still have not been figured out yet. In the present study, it has been observed that TM-derived EVs were a heterogeneous group of nanosized membrane vesicles (30–300 nm) under nanoparticle tracking analysis and transmission electron microscopy. The DiI-labeled EVs could be taken up by RAW 264.7 macrophage cells. Incubation of EVs with macrophages would result in increased expression levels of reactive oxygen species, nitric oxide, and some inflammatory factors including interleukin-1β, interleukin-6, interleukin-10, and tumor necrosis factor. Furthermore, the expression of co-stimulatory molecules (CD80, CD86, and MHC-II) was also increased in macrophages stimulated with EVs. The level of inflammatory factors secreted by macrophages showed a significant decrease when EVs were hydrolyzed by protease, while that of DNA and RNA hydrolase treatment remained unchanged. Subsequently, some virulence factors in EVs including heat shock protein, mannoprotein 1, and peroxidase were determined by liquid chromatography–tandem mass spectrometry. Taken together, our results indicated that the TM-derived EVs could mediate inflammatory response and its protein would play a key role in regulating the function of RAW 264.7 macrophage cells.

## Introduction

*Talaromyces marneffei* (TM, formerly known as *Penicillium marneffei*) is an important pathogenic fungus prevalent in Southeast Asia such as Thailand, northeastern India, Vietnam, and southern China (Vanittanakom et al., 2002; Jiang et al., 2019a). TM is a temperature-dependent diphasic fungus, and its highly contagious filamentous form at 25°C can convert into a pathogenic yeast form at 37°C (Andrianopoulos, 2002). It can cause an opportunistic infection, mainly spreading within immunodeficient populations such as HIV patients in the past few decades (Gugnani et al., 2004). TM infection has become the third most common AIDS-associated infectious disease in the north of Thailand, following pulmonary tuberculosis, and cryptococcal meningitis (Supparatpinyo et al., 1994; Vanittanakom et al., 2006). More than that, with the increased use of immunosuppressants such as anti-IFN-γ in organ transplant rejection reactions and autoimmune diseases, the number of individuals with non-AIDS-related infection caused by TM is on the rise (Chan et al., 2016; Yu et al., 2018). Some specific pathogenic factors have been demonstrated in TM infection so far (Cánovas and Andrianopoulos, 2006; Pongpom et al., 2013; Sapmak et al., 2016; Lau et al., 2018; Feng et al., 2019, 2020). For example, cell wall mannoprotein 1 (MP1p) has been recognized as a key pathogenesis-related protein of TM in the infection process (Sze et al., 2017). There was a significant reduction in organ fungal burden and inflammatory reaction in the TM-MP1p knockout infected mouse model (Woo et al., 2016). However, its specific pathogenic mechanism remains to be further elucidated.

Extracellular vesicles (EVs), a kind of nano-sized lipid bilayer membrane structure, played an essential role in fungal infections such as those of *Cryptococcus neoformans* and *Candida albicans* (Rodrigues et al., 2008; Oliveira et al., 2013; Vargas et al., 2015). Rodrigues et al. (2007) demonstrated that EVs derived from *C. neoformans* carrying the main capsule components glucuronoxylomannan could participate in extracellular capsule reconstruction and could also stimulate mouse-derived macrophages to secrete many kinds of cytokines *in vitro* (Oliveira et al., 2010a). Albuquerque et al. (2008) found that other fungi such as *Candida parapsilosis*, *S. schenckii*, *Histoplasma capsulatum*, and *Saccharomyces cerevisiae* could also release EVs under transmission electron microscopy (TEM). These studies showed that EVs could be secreted from fungi and mediated inflammatory responses; however, whether TM could release EVs and be involved in immune reactions have not been proven yet. Therefore, our research mainly focused on the specific pathogenic effect of TM from a new perspective of EVs.

We have observed that EVs of TM had a typical spherical shape with a diameter of 30 to 300 nm under the nanoparticle tracking analysis (NTA) and TEM for the first time. EVs released by TM could promote the generation of a series of inflammation-related factors and co-stimulatory molecules in RAW 264.7 macrophage cells, but such proinflammatory effect of EVs was weakened when the proteins in the EVs were destroyed. Some virulence factors, including heat shock protein (HSP), MP1p, and peroxidase, were also identified by liquid chromatography–tandem mass spectrometry (LC–MS/MS). In summary, our experimental results showed that TM could secrete EVs, and its proteins further modulated the immune response of RAW 264.7 macrophage cells.

## Materials and Methods

### TM Strain and Growth Conditions

The clinical TM strain was isolated from the blood sample of a patient in Nanfang Hospital in Guangzhou, China. The TM was plated in brain–heart infusion (BHI; Hope Bio-Technology Co., Ltd., Qingdao, China) agar at 37°C for 5–7 days in order to obtain a higher proportion of its yeast cell form (Kummasook et al., 2007; Lu et al., 2019). DNA was extracted from a single pure colony of TM yeast following the Nucleic Acid Extraction Kit (Huaruian Biology, Guangzhou, China) treatment and ITS gene amplification with the primers ITS1 5′-TCCGTAGGTGAACCTGCGG-3′ and ITS4 5′-TCCTCCGCTTATTGATATGC-3′. Subsequently, the PCR products were assayed by agarose gel electrophoresis and sequenced with the Applied Biosystems 3730xl Genetic Analyzer. The ITS gene sequence from this TM was deposited in GenBank (accession no. MN565780). Fungal cells were cultivated in liquid BHI medium for 10 days at 37°C with continuous shaking (150 rpm) in preparation for the isolation of EVs. The study was approved by the Medical Ethics Committee of Nanfang Hospital of Southern Medical University.

### Isolation and Identification of EVs

The isolation of EVs was performed with a modified experimental protocol described previously (Rodrigues et al., 2007). In short, cell-free culture supernatant was collected and followed by sequential centrifugation at 5,000 *g* (15 min, 4°C) and 10,000 *g* (30 min, 4°C). To ensure the removal of the remaining small yeast cells and fungal debris, the supernatant was afterward filtered through a 0.45-μM membrane filter (Merck Millipore, Darmstadt, Germany) and transferred to a new tube. The filtered supernatant was again centrifuged at 5,000 *g* (15 min, 4°C) and 10,000 *g* (30 min, 4°C) and then at 100,000 *g* for 70 min at 4°C in a SW32 Ti rotor (Beckman Coulter, Inc., Brea, CA, United States). The supernatant was discarded, and the pellets were washed two times. The pellets were then resuspended in 100 μl of phosphate-buffered saline (PBS; Gibco Company, Grand Island, MC, United States). In order to obtain EVs of TM of higher purity and quality, OptiPrep (60% iodixanol; Axis-Shield, Norway) density gradient was performed as follows (Roier et al., 2016). We firstly prepared a 50% (w/v) iodixanol working solution by mixing 5 vol of OptiPrep with 1 vol of the 0.25 M sucrose diluents. Then, linear gradients were formed with 40%, 20%, 10%, and 5%, respectively, by diluting the iodixanol working solution with homogenization media (prepared following the manufacturer’s instructions). Resuspended EVs (0.1 ml) were layered on the top of the 5% gradient and spun at 100,000 *g* for 16 h. After density gradient centrifugation, each layer was collected separately and washed with PBS two times by centrifugation at 100,000 *g* for 70 min. Then, the precipitate from four layers was separately collected and analyzed by TEM. Purified EVs were then added to PBS for use in the subsequent experiments or stored at -80°C until further use.

The size distribution of EVs was then determined with the ZetaView NTA (Particle Metrix, Meerbusch, Germany). In our study, the amount of EVs was quantified by the total protein concentration using BCA Protein Assay Kit (Beyotime Biotechnology, Shanghai, China).

The EVs and intact TM yeast cell samples were imaged with TEM (H-7650, Hitachi, Ltd., Tokyo, Japan). TM yeast cells were fixed with glutaraldehyde fixation buffer overnight at 4°C, and the ultrathin section was obtained as described previously (Albuquerque et al., 2008). Moreover, 10-μl drops of EV were added to carbon-coated formvar grids for 20 min, and then the grids were stained with 2% uranyl acetate. The ultrathin section and grids were analyzed under TEM, operating at 80 or 120 kV.

### Internalization of EVs by RAW264.7 Macrophage Cells

The EV samples were stained with DiI (10 μM; Beyotime Biotechnology, Shanghai, China) for 20 min as described previously and then quenched by adding 2 ml of 10% BSA in PBS for 10 min at room temperature (Souza et al., 2019). Cytochalasin D (at a final concentration of 5 μM; Meilun Biotechnology Co., Ltd., Dalian, China) was added to the macrophages 1 h before EVs stimulation and then washed with PBS three times. We made a 0.971-M sucrose solution in advance, and the DiI-labeled EVs solution would be put on top of the sucrose cushion. The DiI-labeled EVs were centrifuged at 100,000 *g* for 70 min and washed three times in PBS. RAW 264.7 macrophage cells (ATCC, Manassas, VA, United States) were plated onto a 24-well plate covered with sterile 15-mm-diameter coverslips (3 × 10^5^ cells per well). After overnight incubation, the culture medium would then be changed to a serum-free medium. Subsequently, the DiI-labeled EVs were incubated with the macrophages for 2 h at 37°C in 5% CO_2_ atmosphere. The RAW 264.7 macrophage cells incubated with unstained EVs served as negative control. The supernatant medium was removed, and EV-treated macrophages were then fixed using 4% paraformaldehyde for 10 min. The cell pellet was washed three times, and the nuclei were labeled with 4′,6-diamidino-2-phenylindole (Beyotime Biotechnology, Shanghai, China) for 3 min. A glass cover was added to the slide with neutral resin after washing. The slides were visualized using a Zeiss LSM 880 with Airyscan confocal microscope (Carl Zeiss, Oberkochen, Germany). Images were acquired using C Plan-Apochromat 63×/1.4 DIC M27 oil immersion objective and processed with the Zeiss analysis software. For all experiments, exosome-free fetal bovine serum (depleted of exosomes by ultracentrifugation for 18 h at 100,000 *g*) or serum-free media were used throughout (Tian et al., 2020).

### Assays of ROS, NO, and Cytokine Production After Stimulation by EVs

RAW264.7 macrophage cells were seeded in six-well plates overnight and then washed with PBS three times, followed by co-incubation with EVs for 12 h. Incubation was performed at 37°C for 20 min after DCFH-DA (10 μM; Beyotime Biotechnology, Shanghai, China) was added, and the stimulated cells were collected using cell scrapers for flow cytometry (FACS Calibur, BD Company, San Diego, CA, United States) under the FITC channel. The EVs were added to a cell culture dish for incubation with RAW264.7 macrophage cells. Nitric oxide (NO) was determined with the Griess reagent (Beyotime Biotechnology, Shanghai, China), and inflammatory factors were assayed with ELISA kit (4A Biotech Co, Ltd, Beijing, China). In order to determine whether the inducible nitric oxide synthase (iNOS) enzyme was specific in the inflammation reaction, S-methylisothiourea sulfate (Beyotime Biotechnology, Shanghai, China), which is highly selective for inhibitor iNOS, was used. The quantitative PCR (qPCR) assay for inflammatory factors was conducted as described below. After RNA was extracted using the TRIzol reagent, nucleic acid concentration and purity were determined with NanoDrop 2000 (Thermo Fisher Scientific Life Sciences, Waltham, MA, United States). RNA samples were amplified with the reverse transcription-PCR (RT-PCR) and qPCR assays utilizing the LightCycler Real-Time PCR 480 System (Roche Diagnostics). The amplification procedure was as follows: 95°C for 30°s, followed by 40 cycles of 95°C for 3 s, 60°C for 30 s, and 95°C, and 5 min for a final extension in one cycle to obtain the melting temperature (*T*_*m*_) value. All qPCR primers were designed with Oligo 7 and are shown in Table 1. In all experiments, lipopolysaccharide (LPS; 1 μg/ml) was used as a positive control. All reagents for RT-PCR and qPCR were from Takara (Biomedical Technology, Beijing, China).

**TABLE 1:** The qPCR primers of reference and target gene-related inflammatory response.

**Gene**	**Primer**	**Sequences**
GAPDH	Forward	5′-AGAACATCATCCCTGCCTCTACT-3′
	Reverse	5′-GATGTCATCATATTTGGCAGGTT-3′
TNF-α	Forward	5′-AACTCCAGGCGGTGCCTATG-3′
	Reverse	5′-TCCAGCTGCTCCTCCACTTG-3′
IL-1β	Forward	5′-AGCTTCAGGCAGGCAGTATC-3′
	Reverse	5′-TCATCTCGGAGCCTGTAGTG-3′
IL-6	Forward	5′-GTCGGAGGCTTAATTACACA-3′
	Reverse	5′-TTCATACAATCAGAATTGCCAT-3′
IL-10	Forward	5′-ACTCTTCACCTGCTCCACTG-3′
	Reverse	5′-GCTATGCTGCCTGCTCTTAC-3′

### Detection of Co-stimulatory Molecules on the Surface of RAW264.7 Macrophage Cells

RAW 264.7 macrophage cells were seeded in six-well plates (5 × 10^5^ cells per well), and then the EVs from TM would be added at 37°C and 5% CO_2_ for 12 h. Blocking of Fc receptors using the antibody CD16/32 was performed to eliminate non-specific background after collecting the RAW264.7 macrophage cells. Then, the cells were stained with anti-CD80 APC, anti-CD86 PE, and anti-MHC-II APC-CY7 antibodies, respectively, for 20 min at 4°C in the dark. Subsequently, the stained cells were washed at least twice with cell staining buffer. Flow cytometry was conducted with LSRFortessa X-20 machine, and its images would be analyzed by using Flowjo software (BD Biosciences, San Jose, CA, United States). All fluorescent antibodies were from BioLegend, Inc. (San Diego, CA, United States). Positive control was LPS (1 μg/ml).

### The Enzymatic Hydrolysis of EVs

Proteinase K, dsDNase, and RNase A (Beyotime Biotechnology, Shanghai, China) were performed for digesting various components in EVs of TM. Proteinase K was added to the EVs suspension, and its action concentration was 2 mg/ml for complete proteolysis of EVs. After proteolysis for 2.5 h at 55°C, the enzymatic reaction was terminated by proteinase inhibitor PMSF (1 mM) for 20 min. For DNA and RNA degradation, dsDNase and RNase A were applied. Then, nuclease was inactivated using a heating metal plate at 65°C for 20 min. Total nucleic acids of 200 μg EVs were extracted using the Nucleic Acid Extraction Kit (Huaruian Biology, Guangzhou, China) and analyzed by gel electrophoresis. Proteins of 2-μg EVs were visualized by silver staining according to the kit instructions (Beyotime Biotechnology, Shanghai, China).

### Proteomic Analysis by LC–MS/MS

Protein digestion and LC–MS/MS were performed as described previously (Jiang et al., 2019b). Briefly, purified vesicles were suspended in 8 M urea and incubated for 20 min at 4°C. The supernatant was collected after centrifugation at 14,000 *g* for 20 min at 4°C. The EVs were then incubated with dithiothreitol (5 mM) at room temperature for 1 h, followed by alkylation with iodoacetamide (10 mM) in the dark for 40 min. The final concentration of urea was then adjusted to 1 M *via* ultrafiltration to exclude the influence of high-concentration urea on follow-up experiments. Then, 4 μg of sequencing-grade trypsin was added to the proteins for overnight incubation at 37°C after the proteins were resuspended in NH_4_HCO_3_ (50 mM), and the reaction was finally terminated by trifluoroacetic acid (0.4%). The proteins were desalted in 10-μg C18 columns and dried in a vacuum centrifuge. Then, 0.5 μg of the peptide mixture suspended in 0.1% formic acid was loaded onto a 2-cm self-packed trap column and then separated on a 75-μm-inner-diameter column with a length of 12 cm over a 60-min gradient at a flow rate of 350 nl/min. The separation was performed on a capillary reverse-phase column connected to a nanoflow high-performance liquid chromatograph instrument (Easy nLC1000 System) coupled to an Orbitrap Fusion mass spectrometer (Thermo Fisher). The Orbitrap Fusion Mass Spectrometry full-scan target value was set to 5 × 10^5^ (mass range m/z 350–1,500), and the maximum injection time was 50 ms. The molecular ion was observed using MS2 scan mode, and only spectra with a charge state of 2–7 were selected for collision-induced dissociation with 30% normalized collision energy. The LC–MS/MS data were searched against *T. marneffei* UniProt database and analyzed using Thermo Proteome Discoverer software (UniProt Consortium, 2018). Trypsin was selected as the cleavage enzyme, with a maximum of two trypsin missed-cleavage parameters. Fixed modifications were carbamidomethylation at cysteine. Oxidation to methionine was selected for variable modification. The main search error was 4.5 ppm, and protein identification false positive rate was set to 1%. All chemicals and reagents were of mass spectrometry grade (Sigma or Thermo Fisher Scientific). The mass spectrometry proteomics data have been deposited to the ProteomeXchange Consortium^1^ *via* the iProX partner repository with the dataset identifier PXD022162 (Ma et al., 2019).

### The Detection of MP1p and Peroxidase in TM-Derived EVs

The MP1p in TM-derived EVs was assayed by ELISA according to the manufacturer’s instructions (Wantai Biological Pharmacy Enterprise Co., Ltd., Beijing, China). The positive cutoff value was calculated as 0.1 plus the mean value of three negative controls. If the value of the negative control was less than 0.05, the value was considered as 0.05. The detection of peroxidase was performed in strict accordance with the kit instructions (Beyotime Biotechnology, Shanghai, China).

### Statistical Analysis

Data analysis was performed by IBM SPSS Statistics (version 25.0; IBM, Armonk, NY, United States). All experiments were repeated three times at different times. Fisher’s exact or chi-square test was applied when appropriate. A *P* value ≤ 0.05 was considered as significant.

## Results

### TM Produces EVs With a Typical Lipid Bilayer Nanostructure

**FIGURE 1:**
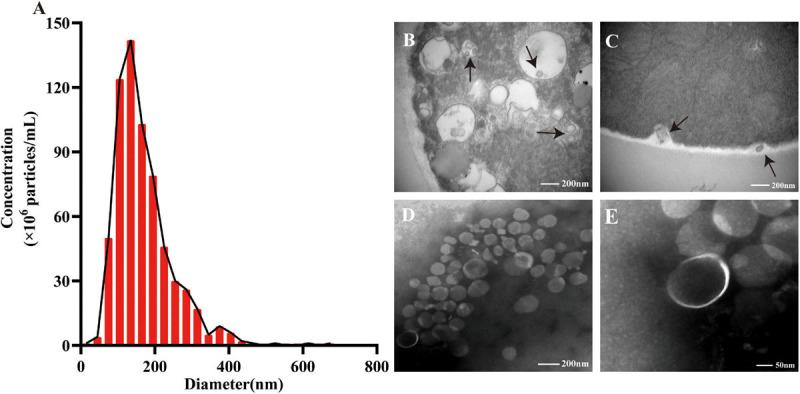
The size distribution and morphology of extracellular vesicles (EVs) secreted by *Talaromyces marneffei* (TM). **(A)** Nanoparticle tracking analysis revealed the size distribution and particle concentration of isolated EVs. **(B)** The EVs were observed inside TM through transmission electron microscopy (TEM; black arrow). **(C)** It showed the moment of TM yeast cells releasing EVs (black arrow). **(B,C)** Scale bar = 200 nm. **(D,E)** TEM identified the purified EVs’ morphology with a cup-shaped structure. **(D)** Scale bar = 200 nm. **(E)** Scale bar = 50 nm.

Our experiment indicated that purified EVs were mainly localized in the 20% fraction since the density of EVs was different from those of other impurities (Supplementary Figure 1). To investigate the particle count and size distribution of the EVs secreted by TM, the ZetaView NTA was utilized, and it showed that the diameter of these vesicles was 169.72 ± 78.77 nm (Figure 1A). The generation of EVs inside the TM yeast cells was observed, and the moment when TM released the vesicles through the thick cell wall has also been shown under TEM (Figures 1B,C). The morphology characteristic of EVs derived from TM culture supernatant was confirmed again, and its typical cup-shaped vesicular structure was shown (Figures 1D,E). To our knowledge, our study’s results have shown for the first time that TM could secrete EVs.

### The EVs of TM Were Taken Up by RAW264.7 Macrophage Cells

**FIGURE 2:**
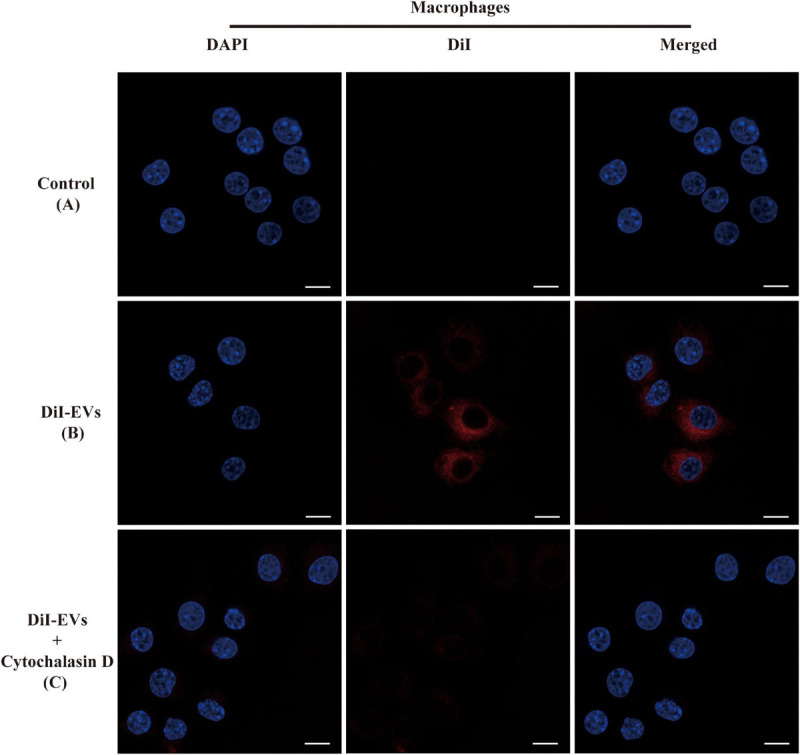
Immunofluorescence images showed the process of RAW264.7 macrophage cells uptaking extracellular vesicles (EVs). The RAW264.7 macrophage cells were incubated with unstained EVs **(A)** and labeled vesicles **(B)** after 2 h of co-incubation. **(C)** RAW264.7 macrophage cells were incubated with actin polymerization inhibitors cytochalasin D for 2 h before the addition of EVs. EVs were labeled with DiI staining (red). Cell nuclei were counterstained with 4′,6-diamidino-2-phenylindole (blue). The fluorescent images are representative of three independent experiments (scale bar = 10 μm).

The ability of RAW264.7 macrophage cells in effectively uptaking EVs was examined through fluorescent signal detection. To test whether EVs of TM could go across the cellular membrane *via* passive diffusion or active transport, cytochalasin D was used to disrupt F-actin fibers in RAW264.7 macrophage cells, inhibiting both its cell motility and phagocytosis of EVs (Bielska et al., 2018). Cytochalasin D led to a significant decline in EV endocytosis compared with the non-treated group after 2 h of incubation with the macrophages (Figure 2).

### ROS, NO, and Cytokine Production Were Regulated by EVs of TM in RAW264.7 Macrophage Cells

**FIGURE 3:**
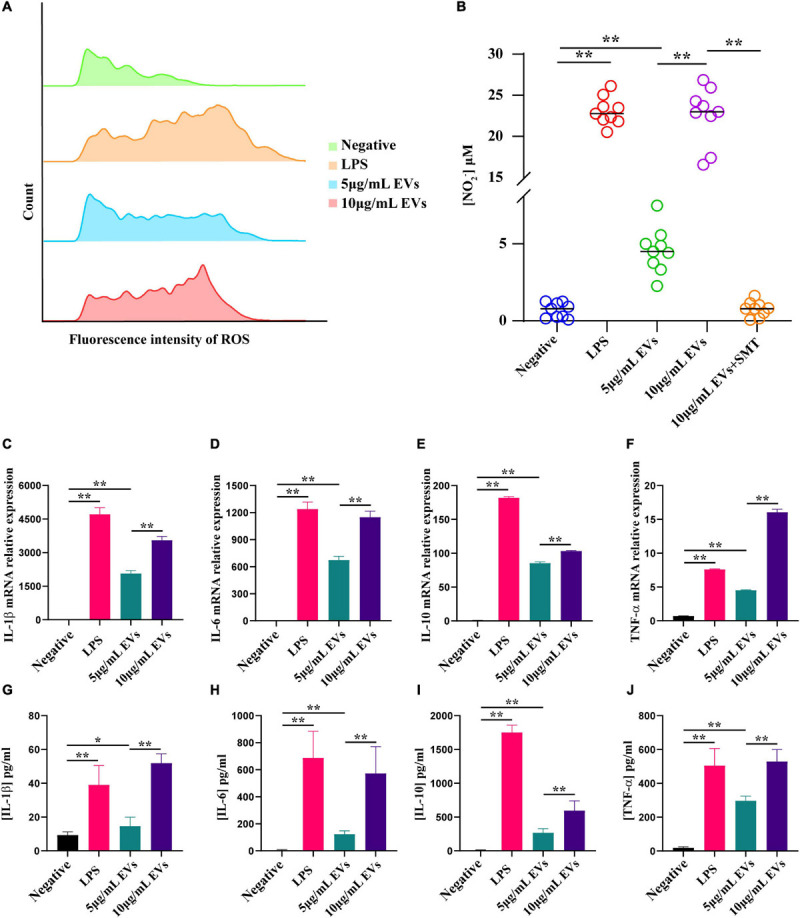
Determination of reactive oxygen species (ROS), nitric oxide (NO), and inflammatory factor production after co-incubation of RAW264.7 macrophage cells with various concentrations of extracellular vesicles (EVs) derived from *Talaromyces marneffei* (TM). **(A)** ROS production was detected by flow cytometry after co-incubation of RAW264.7 macrophage cells with EVs for 12 h. From top to bottom, the groups were negative (green), lipopolysaccharide (LPS; orange), 5 μg/ml EVs (blue), and 10 μg/ml EVs (pink), respectively. **(B)** The change of NO was determined using the Griess reagent. The groups were negative, LPS, 5 μg/ml EVs, 10 μg/ml EVs, and 10 μg/ml EVs + SMT, respectively. **(C–F)** The RNA was isolated for qPCR after 24 h of stimulation of TM-derived EVs. The bar chart above shows the mRNA level of inflammatory factors. **(G–J)** The supernatants from the macrophages stimulated with EVs for 24 h were collected, and the cytokine levels were measured by ELISA. The bar chart below represents the ELISA result. *P < 0.05, **P < 0.001. LPS, positive. *n* = 3 independent experiments; Student’s *t*-test.

Oxidative stress factors, especially reactive oxygen species (ROS) and NO, were reported to play a central role in the immune process of host cell defensing against TM (Cogliati et al., 1997). The experimental finding suggested that ROS and NO were significantly increased in RAW264.7 macrophage cells after incubation with different concentrations of EVs for 24 h compared to control (Figures 3A,B). There were three isoenzymes of nitric oxide synthase (NOS), including nNOS in neurons, iNOS in macrophages, and eNOS in endothelial cells (De Felice et al., 2010). To verify the specificity of iNOS in RAW264.7 macrophage cells stimulated with EVs, S-methylisothiourea sulfate (SMT), the iNOS-selective inhibitor, was used (Pott Godoy et al., 2008). The expression level of NO has markedly decreased since SMT was added. Subsequently, the gene and protein expression levels of various inflammatory factors were determined by qPCR and ELISA, respectively, after EVs were added to the RAW264.7 macrophage cells for 24 h. Notably, the results showed that interleukin-1β (IL-1β), interleukin-6 (IL-6), interleukin-10 (IL-10), and tumor necrosis factor (TNF-α) were significantly up-regulated following the interaction between RAW264.7 macrophage cells and EVs derived from TM (Figures 3C–J). The experiment was conducted to confirm that EVs derived from TM could promote the inflammatory response by secreting relevant cytokines.

### TM-Derived EVs Regulated the Expression of Cell Surface Co-stimulatory Molecules in RAW 264.7 Macrophage Cells

**FIGURE 4:**
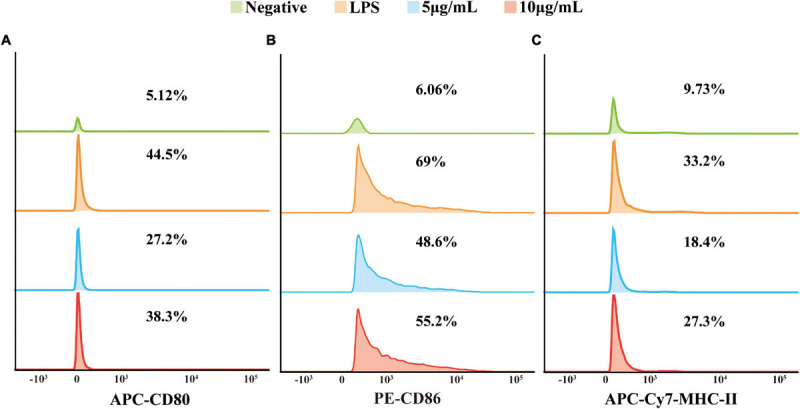
The expression of co-stimulatory molecules CD80, CD86, and MHC-II was examined with flow cytometry. The ordinate was the number of cells, and the abscissa was the fluorescence intensity. From top to bottom: negative, lipopolysaccharide (positive), 5 μg/ml EVs, and 10 μg/ml EVs. From left to right: CD80 **(A)**, CD86 **(B)**, and MHC-II **(C)**. The experiments were repeated for three independent times.

It was hypothesized that the EVs of TM could also be involved in T cell activation since stimulated RAW264.7 macrophage cells released a series of inflammatory mediators. In order to verify our speculation, RAW264.7 macrophage cells were stimulated with EVs secreted by TM and co-stimulatory molecules CD80 (B7-1), CD86 (B7-2), and MHC-II were detected by flow cytometry. Our data showed that the gene expression levels of CD80, CD86, and MHC-II were indeed increased (Figure 4).

### The Protein Function and Composition of TM-Derived EVs Were Investigated

**FIGURE 5:**
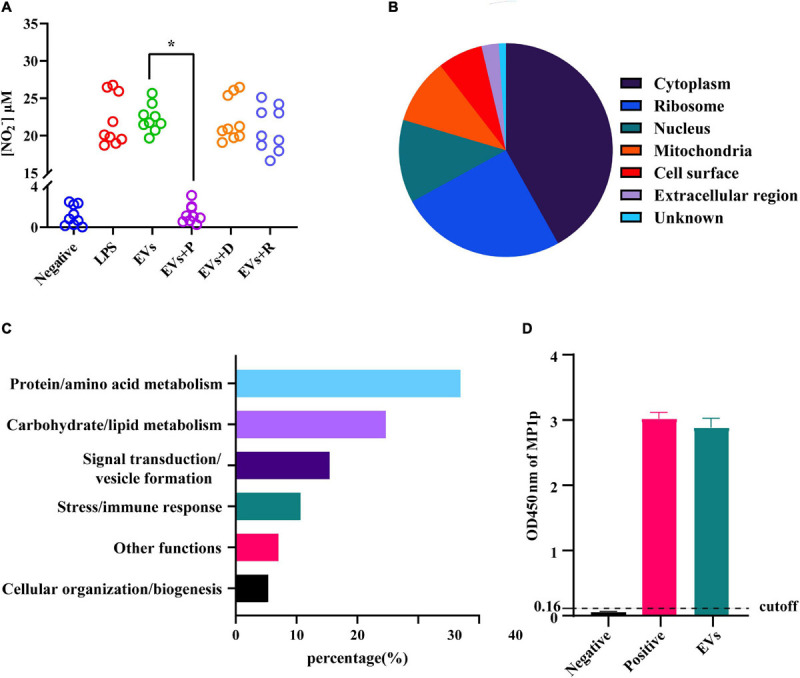
Detection of nitric oxide (NO) in different extracellular vesicles (EV) treatment groups and functional classification and subcellular localization of proteins in EVs derived from *Talaromyces marneffei* (TM). **(A)** The NO levels in the supernatants were measured after EVs were added to RAW264.7 macrophage cells for 24 h. The three EV-treated groups are as follows: P, protease; D, DNA hydrolase; and R, RNA hydrolase. **(B)** The pie graphs approximately reflected the distribution of protein localization in TM-derived EVs, and different colors represented their different localization. **(C)** The vertical axis was the functional classification, and the horizontal axis was the percentage of every category of protein on the annotation. **(D)** The ordinate was the optical density (OD) value of MP1p ELISA among different groups. The result was positive if the OD value was greater than the cutoff value, and three independent experiments were performed. *P < 0.05.

It is well known that EVs contain various biomolecules, including specific proteins and nucleic acids. In order to figure out the main bioactive components of EVs, the following enzyme-related experiments were designed. Those enzymes were used for the degradation of different ingredients formerly shown to be enriched in EVs secreted from other fungi. The degradation of DNA or RNA in EVs did not impair their ability to activate the RAW264.7 macrophage cells. On the contrary, the secretion level of NO in RAW264.7 macrophage cells was shown to be significantly decreased after protein digestion (Figure 5A), and the same trend could also be seen in some inflammation factors, including IL-1β, IL-6, IL-10, and TNF-α (Supplementary Figure 2). The EVs derived from TM were subjected to LC–MS/MS for further understanding of the specific composition of proteins within the EVs. A total of 394 proteins have been identified, and more information, including protein accession ID, molecular weight, and isoelectric point, could be found in Supplementary Table 1. According to the given mass spectral data, there were some proteins similar to that of EVs derived from *C. albicans* and *C. neoformans* (Rodrigues et al., 2008; Vargas et al., 2015), which were also found in the exosome database, confirming the purity of the vesicles. The functional and the subcellular location classification of proteins were carried out according to the Uniprot and Gene Ontology database. A total of 67% of proteins were predominantly located in the cytoplasm and ribosome, and the location of the remaining proteins included the cell surface, mitochondria, nucleus, extracellular region, and unknown (Figure 5B). Then, the proteins were sorted into six categories based on their functions, including protein/amino acid metabolism, stress/immune response, signal transduction/vesicle formation, carbohydrate/lipid metabolism, cellular organization/biogenesis, and other functions (Figure 5C). Most of the proteins were closely related to other members in the protein–protein interaction network of TM-derived EVs, and the protein/amino acid metabolism group was in the center of the network (Supplementary Figure 3). In addition, some proteins of EVs have been found to be associated with fungal virulence and host immune response in TM infection, such as HSP, MP1p, and peroxidase. We next verified the MP1p and peroxidase in our mass spectrometry results in a second assay. The experimental results demonstrated that MP1p did exist in TM-derived EVs (Figure 5D), while peroxidase did not exist (data not shown).

## Discussion

*Talaromyces marneffei* is one of the most important fungi which could cause opportunity infection in the immunocompromised population in Southeast Asia. TM infection has similar non-specific clinical signs with *Mycobacterium tuberculosis* and is always misdiagnosed at the early stage of TM infection. TM could participate in the host–pathogen interaction through direct contact or the secretion of bioactive molecules (Macia et al., 2019). There were also several reports on some virulence factors of TM such as HSP, antioxidant enzymes, MP1p, and nutritional metabolism-related enzymes (Kummasook et al., 2007; Vanittanakom et al., 2009; Woo et al., 2010; Sze et al., 2017). However, the specific pathogenesis of TM has not been fully figured out, and it needed to be investigated from a new perspective.

Extracellular vesicles could act as a carriage with a lipid bilayer-enclosed structure to encapsulate numerous active molecules biologically, and taking EVs from fungi as example, it could transport some important molecules to the extracellular environment, thus mediating further processes. Specifically speaking, the intracellular proliferation rate of non-epidemic *Cryptococcus gattii* strains could be significantly improved by EVs of epidemic strains (Bielska et al., 2018). It was also reported that other fungi such as *H. capsulatum*, *Malassezia sympodialis*, and *Paracoccidioides brasiliensis* could secrete EVs loaded with some active molecules (Vallejo et al., 2012; da Silva et al., 2016; Vallhov et al., 2020). However, it remained unclear whether TM could secrete EVs. The yeast form of TM was selected as the study object since its hyphae form could be finally converted to yeast form primarily within macrophages of the human body.

In this study, the TM yeast-derived EVs were isolated by differential centrifugation and further purified through OptiPrep density gradient ultracentrifugation. Much more highly purified EVs could ensure the repeatability of the experiments. The NTA and TEM showed that the mean diameter of EVs was 169.72 ± 78.77 nm, and their structures were typical of cup-shaped vesicles, which were similar to that of *S. cerevisiae* and *C. neoformans* (Albuquerque et al., 2008; Oliveira et al., 2010b). It is worth noting that a multi-vesicular body structure, the precursor for EV synthesis (Supplementary Figure 4), was observed, and the moment of TM releasing EVs has also been visualized by TEM.

To investigate the specific function of EVs derived from TM, a co-culture model of RAW264.7 macrophage cells with DiI-labeled EVs was built, and it showed that macrophages could intake EVs but could not with pretreatment of actin polymerization inhibitor cytochalasin D, which confirmed that the vesicle internalization of macrophage was an active process but not a passive movement. Up until now, there were several literatures indicating that the EVs of some fungi, including *C. albicans* and *C. neoformans*, could promote macrophage activation and the production of a vast array of inflammatory chemokines (Oliveira et al., 2010a; Vargas et al., 2015). For instance, the expression levels of NO, IL-10, IL-12, and TNF-α in macrophage and its surface expression of co-stimulatory molecule CD86 and MHC-II in DC cells would increase significantly in the model of co-incubation with EVs derived from *C. albicans*. However, the effect of EVs derived from TM on macrophage still has not been reported yet. For the first time, our present report has confirmed that EVs could be secreted from TM and phagocytized by RAW264.7 macrophage cells. Subsequently, the function of EVs in cellular inflammatory response was explored. NO has been proven to participate in cellular anti-TM immunity (Cogliati et al., 1997; Cao et al., 2009) and could be eventually converted to reactive nitrogen species (RNS). RNS and ROS were highly toxic to fungal cells, which could lead to structural disintegration and final death of TM in macrophages (Pongpom et al., 2017). Our study revealed that the EVs of TM could significantly promote NO and ROS secretion in RAW264.7 macrophage cells, which indicated TM as an intracellular fungus that could effectively trigger oxidative stress in macrophages through secretory vesicles. The application of the inhibitor SMT proved that the increase of NO secretion in macrophages incubated with TM-derived EVs was induced specifically by iNOS. Oxidative stress was closely associated with inflammation response in regulating immune responses. In addition, the patients infected with TM have previously been reported to have higher TNF-α, IL-1β, IL-12, and IL-10 levels *in vivo*, and the infection of TM enabled the enhancement of the production of cytokines such as TNF-α, IL-1β, IL-6, and IL-12 by stimulating macrophages *in vitro* (Dai et al., 2017; Dong et al., 2019). Therefore, a series of cytokines from macrophages was also measured, and the results have confirmed that EVs could enhance the secretion of IL-1β, IL-6, IL-10, and TNF-α in RAW264.7 macrophage cells, while there was no change in the IL-12 expression level. The possible reason for this difference may be the different infection processes of TM and EVs in macrophages, and further research is needed. Acting as professional antigen-presenting cells, macrophage cells were also known to play an important role in the regulation of intercellular communication, in which co-stimulatory molecules were thought to have indispensable roles in inducing T-cell activation, antigen processing, and presentation (Vargas et al., 2020). We have shown previously that macrophage cells stimulated with EVs could produce various inflammatory factors, which indicated that TM-related antigens on vesicles might be presented on the cell surface and regulate the immune cell activation. The results in our study showed that the expression levels of co-stimulatory molecules CD80, CD86, and MHC-II on the surface of RAW264.7 macrophage cells likewise increased after co-incubation with EVs of TM, similar to *C. albicans* and *Leishmania donovani* (Silverman et al., 2010; Vargas et al., 2015). In a word, RAW264.7 macrophage cells could interact with EVs of TM and promoted the secretion of a series of inflammatory cytokines and co-stimulatory molecules.

The change in immune stimulation of EVs was observed after protein, DNA, and RNA were being hydrolyzed, respectively, for investigating the main functional components of EVs. The experiment showed that the immunostimulatory effect of EVs was significantly weakened with protease pretreatment, but not in pretreatment with dsDNA and RNase A hydrolase. DNase and RNase were very specific for the degradation of nucleic acid, and they had no effect on the protein (Supplementary Figure 5). In summary, we concluded that the proteins in EVs were the major regulator of inflammatory response during the interaction between EVs and RAW264.7 macrophage cells. To further investigate its protein composition, EVs derived from TM were further analyzed by LC–MS/MS and classified to work out their functions and cellular localization. A total of 394 proteins were identified, which revealed that most of them were involved in protein/amino acid metabolism and signal transduction and that the proteins of EVs were localized mainly in the cytoplasm, ribosome, and nucleus. There were also some proteins about vesicle synthesis and secretion, such as HSP, GPI-anchored proteins, Ras GTPase, histones, and ribosomal proteins. Some proven or putative virulence factors, including HSP, MP1p, and peroxidase, were also identified in the proteome of TM-derived EVs. The HSP could not be further confirmed currently because of the lack of specific antibodies. MP1p was validated using the detection kit, but no peroxidase activity was detected. It could be explained by the fact that EVs might carry a peroxidase fraction and lack the complete catalytic portion. At the moment, we can thus conclude that TM-derived EVs may carry MP1p involved in immunoreactions and stress response. However, MP1 gene knockout experiments have not been performed in the present study, and its role in macrophage-stimulated EVs would be confirmed in subsequent research.

As an important carrier containing a variety of molecules, EVs played a vital role in intercellular communication. This was the first time to confirm that the EVs of TM existed and could participate in immune responses by activating macrophages, which provided us a new perspective to further explore the mechanism of TM infection.

## Data Availability Statement

The datasets presented in this study can be found in online repositories. The names of the repository/repositories and accession number(s) can be found below: ProteomeXchange (accession: PXD022162) and NCBI GenBank (accession: MN565780).

## Ethics Statement

The studies involving human participants were reviewed and approved by Medical Ethics Committee of Nanfang Hospital of Southern Medical University. The patients/participants provided their written informed consent to participate in this study.

## Author Contributions

BY, JW, HJ, HL, XH, LZ, and QW: conceptualization and writing—review and editing. BY, JW, HL, ZO, AU, YH, JC, and XL: investigation. BY, JW, HJ, ZO, AU, and YH: data analysis. BY and XL: writing—original draft preparation. XH, LZ, and QW: project administration. All authors have read and agreed to the published version of the manuscript.

## Conflict of Interest

The authors declare that the research was conducted in the absence of any commercial or financial relationships that could be construed as a potential conflict of interest.

## Footnotes

1 http://proteomecentral.proteomexchange.org

## Abbreviations

EVs: extracellular vesicles
TM: *Talaromyces marneffei;*
TEM: transmission electron microscopy
ROS: reactive oxygen species
NO: nitric oxide
HSP: heat shock protein
MP1: mannoprotein 1
GXM: glucuronoxylomannan
NTA: nanoparticle tracking analysis
LC–MS/MS: liquid chromatography–tandem mass spectrometry.
