# Supplementary figures and images for: Extracellular Vesicles Derived From Talaromyces marneffei Yeasts Mediate Inflammatory Response in Macrophage Cells by Bioactive Protein Components
Source: Front Microbiol. 2021 Jan 8;11:603183. doi: 10.3389/fmicb.2020.603183 (PMC7819977; doi:10.3389/fmicb.2020.603183)

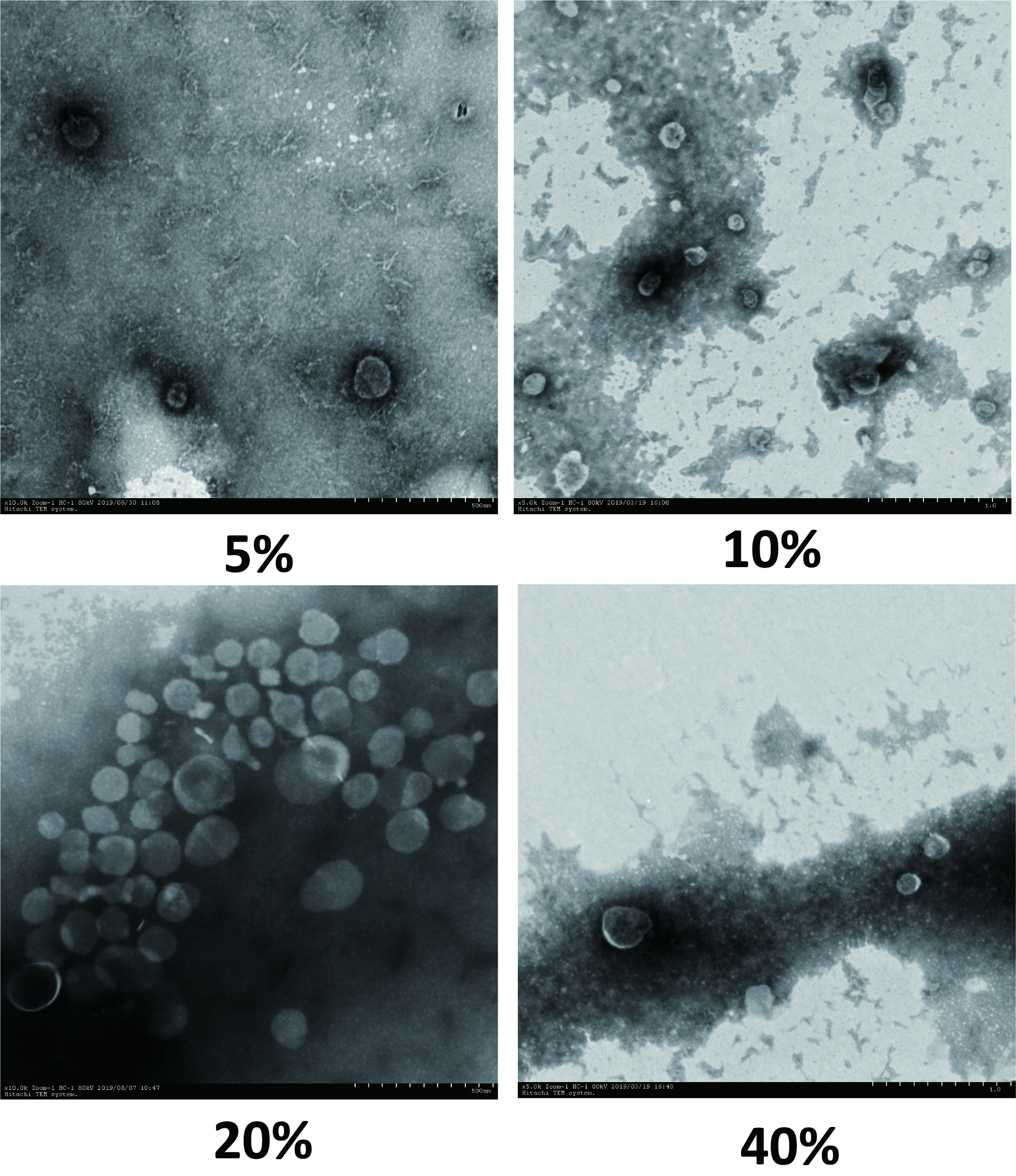

Supplement: Supplementary file 1 [file Data_Sheet_1.ZIP › additional files/Figure S1.tif]

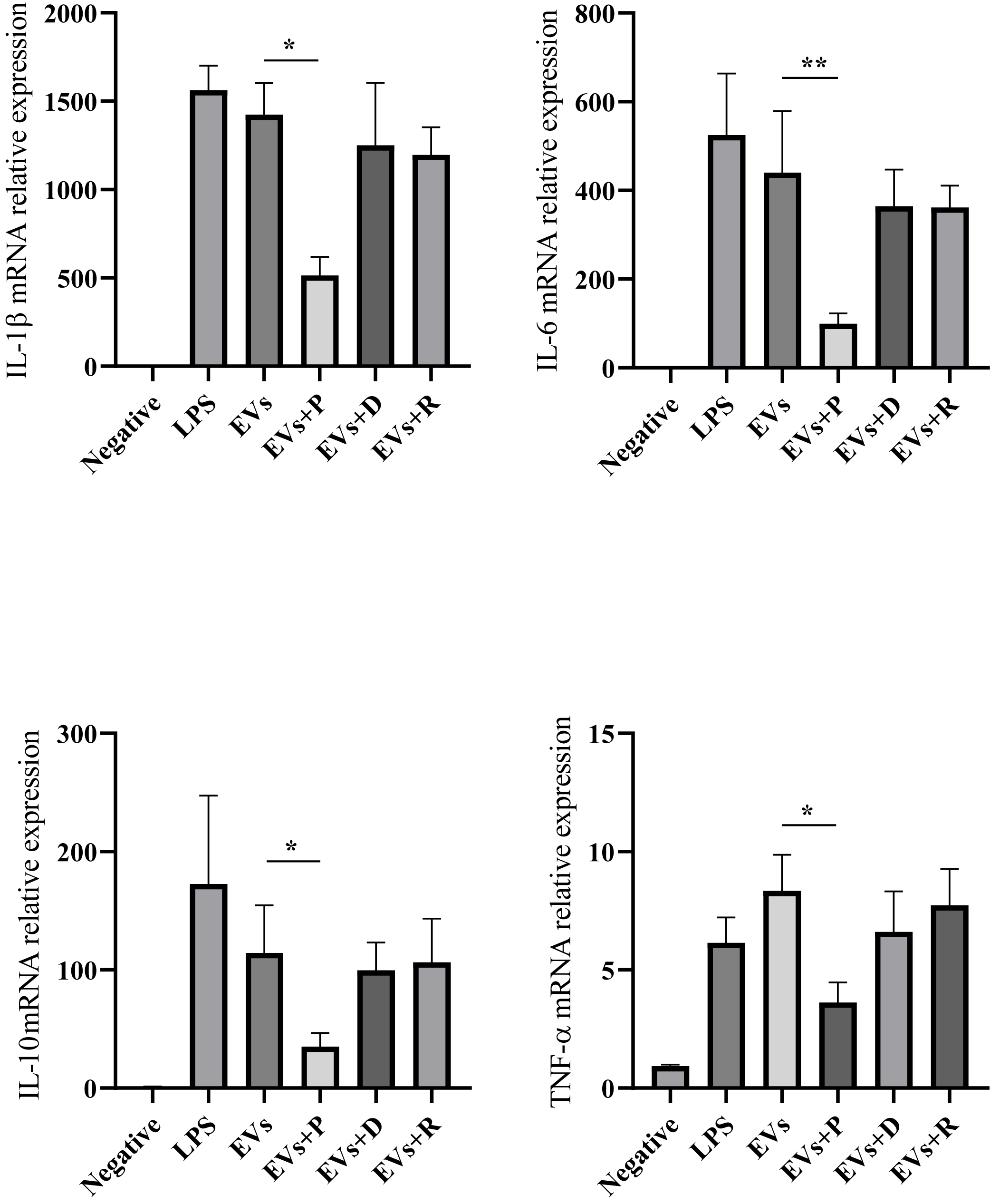

Supplement: Supplementary file 1 [file Data_Sheet_1.ZIP › additional files/FIgure S2.tif]

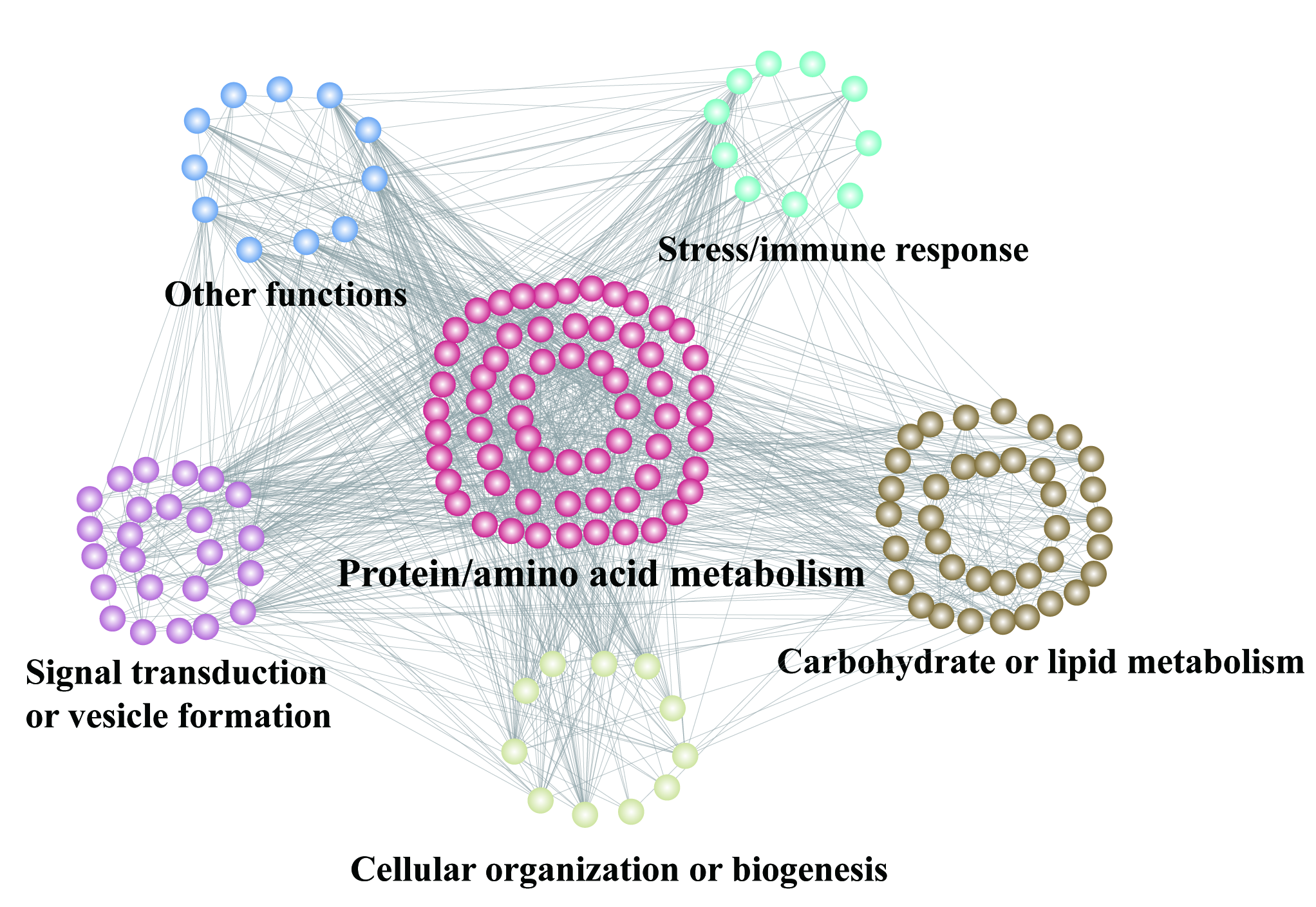

Supplement: Supplementary file 1 [file Data_Sheet_1.ZIP › additional files/Figure S3.tif]

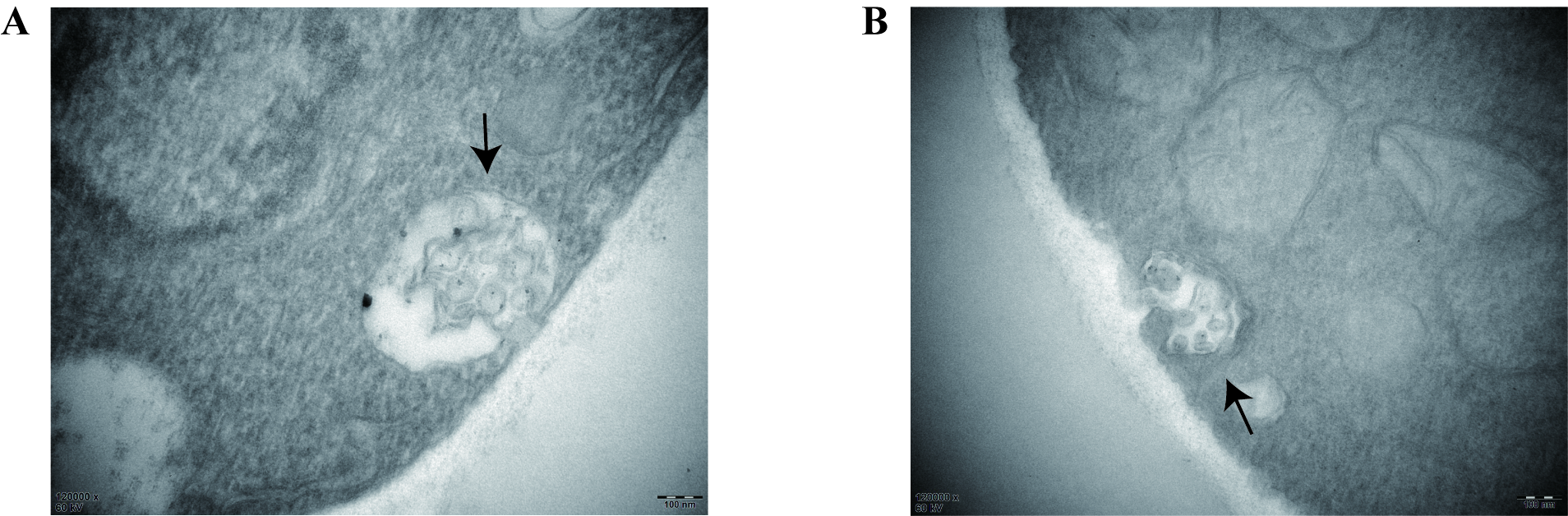

Supplement: Supplementary file 1 [file Data_Sheet_1.ZIP › additional files/Figure S4.tif]

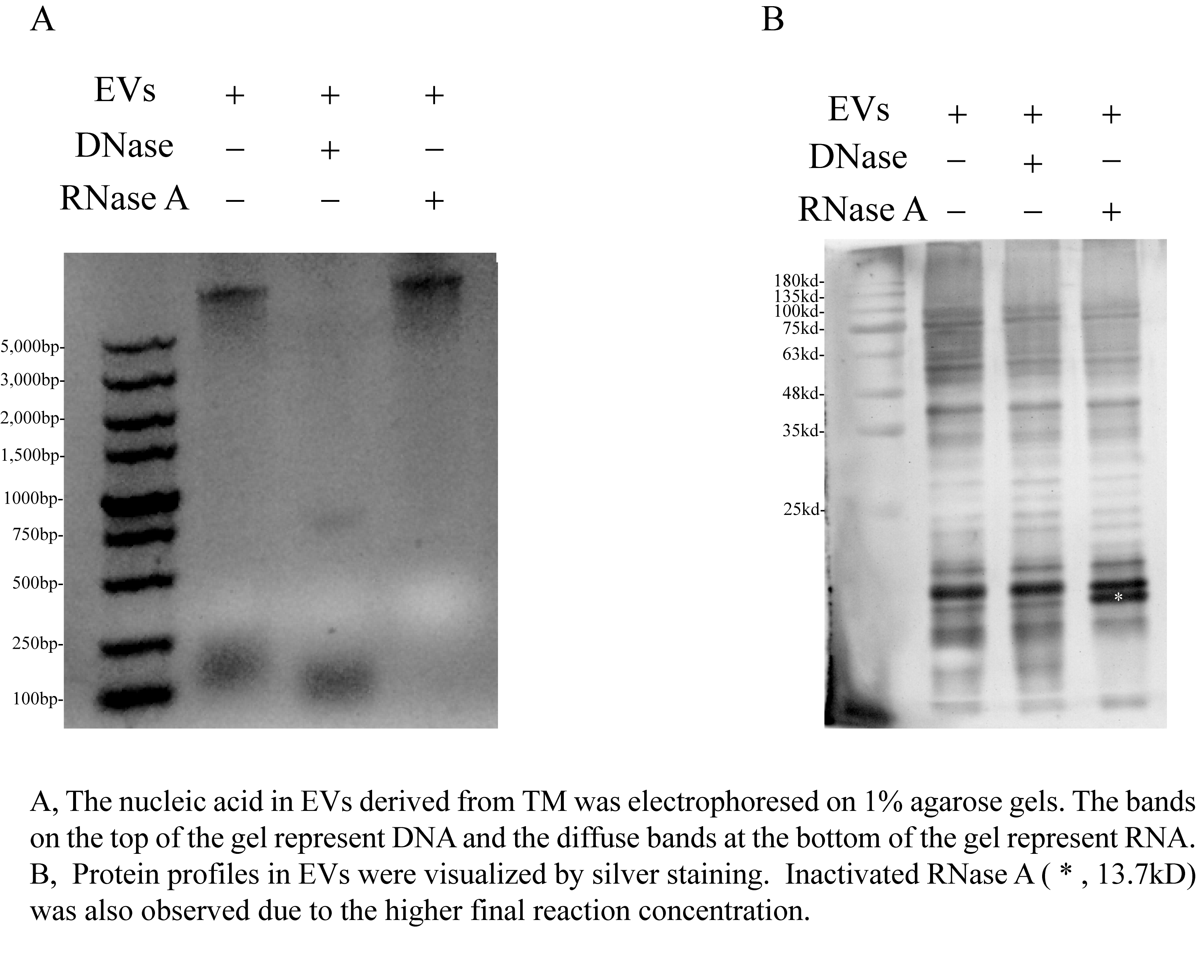

Supplement: Supplementary file 1 [file Data_Sheet_1.ZIP › additional files/Figure S5.tif]
